# The Antibacterial Activity of Thymol Against Drug-Resistant *Streptococcus iniae* and Its Protective Effect on Channel Catfish (*Ictalurus punctatus*)

**DOI:** 10.3389/fmicb.2022.914868

**Published:** 2022-06-06

**Authors:** Lizi Yin, Chao Liang, Wenyan Wei, Shuanghui Huang, Yongqiang Ren, Yi Geng, Xiaoli Huang, Defang Chen, Hongrui Guo, Jing Fang, Huidan Deng, Weimin Lai, Shumin Yu, Ping Ouyang

**Affiliations:** ^1^Department of Basic Veterinary, College of Veterinary Medicine, Sichuan Agricultural University, Chengdu, China; ^2^Aquaculture Research Institute, Chengdu Academy of Agriculture and Forestry Sciences, Chengdu, China; ^3^Department of Aquaculture, College of Animal Science and Technology, Sichuan Agricultural University, Chengdu, China

**Keywords:** thymol, *Streptococcus iniae*, antibacterial mechanism, transcriptomic, channel catfish (*Ictalurus punctatus*), protective effect

## Abstract

*Streptococcus iniae* is a zoonotic pathogen, which seriously threatens aquaculture and human health worldwide. Antibiotics are the preferred way to treat *S. iniae* infection. However, the unreasonable use of antibiotics leads to the enhancement of bacterial resistance, which is not conducive to the prevention and treatment of this disease. Therefore, it is urgent to find new efficient and environmentally friendly antibacterial agents to replace traditional antibiotics. In this study, the antibacterial activity and potential mechanism of thymol against *S. iniae* were evaluated by electron microscopy, lactate dehydrogenase, DNA and protein leakage and transcriptomic analysis. Thymol exhibited potent antibacterial activity against *S. iniae in vitro*, and the MIC and MBC were 128 and 256μg/mL, respectively. SEM and TEM images showed that the cell membrane and cell wall were damaged, and the cells were abnormally enlarged and divided. 2MIC thymol disrupted the integrity of cell walls and membranes, resulting in the release of intracellular macromolecules including nucleotides, proteins and inorganic ions. The results of transcriptomic analysis indicated that thymol interfered with energy metabolism and membrane transport, affected DNA replication, repair and transcription in *S. iniae*. *In vivo* studies showed that thymol had a protective effect on experimental *S. iniae* infection in channel catfish. It could reduce the cumulative mortality of channel catfish and the number of *S. iniae* colonization in tissues, and increase the activities of non-specific immune enzymes in serum, including catalase, superoxide dismutase, lysozyme and acid phosphatase. Taken together, these findings suggested that thymol may be a candidate plant agent to replace traditional antibiotics for the prevention and treatment of *S. iniae* infection.

## Introduction

*Streptococcus iniae* is a beta-haemolytic, facultative anaerobic Gram-positive bacterium distributed worldwide. It has a wide range of pathogenicity to a variety of freshwater and marine fish, such as *Oreochromis niloticus, Lates calcarifer, Amazon catfish, Acipenser baerii* and *Oncorhynchus mykiss* (Deng et al., 2015; Lukkana et al., 2016; Tavares and de Queiroz, 2018; Daneshamouz et al., 2019; Khang et al., 2019). The disease is characterized by enteritis (Piamsomboon et al., 2021), generalized septicaemia and meningoencephalitis (Chen et al., 2007), and causes a high mortality rate of about 50% (Park et al., 2007; Nawawi et al., 2008; Rahmatullah et al., 2017). In recent years, *S. iniae* has become one of the most important fish pathogens, causing hundreds of millions in losses to aquaculture every year (Agnew and Barnes, 2007; Chen et al., 2012). In addition, *S. iniae* is also considered as a zoonotic pathogen like other *Streptococcus* spp., especially for people with low immunity or associated with processing fresh fish (Lim et al., 2021). In humans, *S. iniae* infection often causes bacteraemic cellulitis, which subsequently leads to serious complications including arthritis, meningitis, endocarditis and osteomyelitis (Fulde and Valentin, 2013). Antibiotics were an important way to treat and control *S. iniae* infection (Agnew and Barnes, 2007). Most *S. iniae* strains isolated from humans have been shown to be sensitive to β-lactams, macrolides, quinolones, and vancomycin, and penicillin is the drug of choice for the treatment of *S. iniae* infections in mammals (Facklam et al., 2005). However, unreasonable use of antibiotics has contributed to the development of *S. iniae* resistance. For example, *S. inia*e isolated from hybrid tilapia in Malaysia was resistant to streptomycin, ampicillin, penicillin, and erythromycin (Rahmatullah et al., 2017). *Streptococcus iniae* strains were highly resistant to megamycin, polymyxin B and synovomin in Sichuan Province of China (Feng et al., 2021). In addition, the accumulation of antibiotics in the environment and fish poses a potential risk to human and public health (Lim et al., 2021). Therefore, it is necessary and urgent to develop natural alternatives of antibiotics to control drug-resistant *S. iniae* infections, especially in aquaculture.

In recent years, studies have mainly focused on natural botanical active ingredients due to their excellent antibacterial activities, safety, and environmental friendliness. Thymol is the main component of thyme essential oil from *Thymus* and *Lamiaceae* plants, and this monoterpene derivative has a wide range of uses in many industries including medical, food, pesticide and perfume (Rivas et al., 2010; Meeran et al., 2017). A large number of experimental results *in vitro* and *in vivo* have confirmed that thymol has antioxidant, anti-inflammatory, anti-cancer and immunomodulatory effects, which makes thymol have a good effect in the treatment of nervous system, respiratory system and cardiovascular system diseases (Meeran et al., 2017; Salehi et al., 2018). In addition, many reports have demonstrated that the thymol have a potent inhibitory activity on both gram-positive and gram-negative pathogens, including *Salmonella* (Zhou et al., 2007), *Aeromonas hydrophila* (Liang et al., 2021), *Listeria monocytogenes* (Ilhak and Guran, 2014), *Streptococcus pneumoniae* (Lakis et al., 2012) and *Staphylococcus aureus* (Yuan et al., 2019).

With this background in mind, due to the lack of studies on natural plant ingredients replacing traditional antibiotics in the treatment of *S. iniae* infection, the present study aims to investigate the antibacterial mechanism of thymol against *S. iniae* isolated from sturgeon, and to study the prevention potential of thymol on infected channel catfish.

## Materials and Methods

### Bacterial Strain and Chemicals

Drug-resistant *S. iniae* strain HT (Genbank: MK973068) was isolated from infected Dabry's Sturgeon (Acipenser dabryanus) and cultured in Brain Heart Infusion (BHI) broth or agar at 28°C. Thymol (>98% HPLC purity; CAS no. 89-83-8) was bought from Zhanyun Chemical Co., Ltd. (Shanghai, China) and dissolved in dimethyl sulfoxide (DMSO, Sigma, St. Louis, MO) to obtain a stock solution (40.96 mg/mL). Phosphate buffered saline, pre-mixed powder was purchased from Sangon Biotech Co., Ltd. (Shanghai, China). The drug-susceptible discs were purchased from Hangzhou Taihe Microbiological Reagent Co., Ltd. (Hangzhou, China). The Bicinchoninic acid (BCA) assay kit was purchased from Solarbio Technology Co., Ltd. (Beijing, China). The lactate dehydrogenase (LDH) (A020-2-2), superoxide dismutase (SOD) (A001-3-2), catalase (CAT) (A007-1-1), lysozyme (LZM) (A050-1-1) and acid phosphatase (ACP) (A060-1-1) assay kits were purchased from Nanjing Jiancheng Bioengineering Institute (Nanjing, China). ChamQ Universal SYBR qPCR Master Mix and HiScript III RT SuperMix for qPCR (+gDNA wiper) were purchased from Vazyme Biotech Co., Ltd. (Nanjing, China).

### Fish and Feeding Conditions

One hundred and fifty-five healthy channel catfish with an average weight of 60 ± 5.0 g were obtained from a commercial fish farm in Sichuan. All experimental animals were adapted to standardized circulating water environmental conditions (temperature = 26 ± 1°C; dissolved oxygen = 6.5 ± 0.5 mg/L) 1 week before infection, and 5 fish were subjected to microbiological analysis to verify that all fish were negative for *S. iniae* (Zhang et al., 2016). All animal feeding and handling procedures were approved by the Animal Care and Use Committee of Sichuan Agricultural University, and the experimental protocols were performed in accordance with the Animal Experiment Guidelines of Sichuan Agricultural University, under permit number LC-2019403130.

### Antibiotic Susceptibility Test

The Kirby-Bauer disc diffusion method (K-B-method) was used to detect the drug resistance of the strain in accordance with the National Committee for Clinical Laboratory Standards Institute Guidelines (CLSI) (Rahmatullah et al., 2017; Feng et al., 2021). The concentration of *S. iniae* was adjusted to 1.5 × 10^8^ CFU/mL according to the McFarland's turbidimetric method, which was coated on the surface of BHI agar with a sterile cotton swab. Afterwards, the drug-susceptible discs were marked and affixed to the plate. After 24 h incubation at 28°C, the diameter of the inhibition zone was measured. The results obtained were interpreted according to the recommended clinical breakpoint of the standard in use (Gajic et al., 2022).

### Antibacterial Activity of Thymol Against *S. iniae* and Growth Curve Assay

The antibacterial activity of thymol was studied by determining the minimum inhibitory concentration (MIC) and the minimum bactericidal concentration (MBC) of thymol against *S. iniae* HT strain (Zainol et al., 2013). MICs were determined by gradient dilution method. Briefly, *S. iniae* was co-cultured with thymol diluted to various concentrations (1,024, 512, 256, 128, 64, 32, 16, and 8 μg/mL) in BHI broth in a 96-well plate at 28°C, and MIC mean the lowest concentration of thymol in the wells without bacterial growth after 24 h. Further, the mixture in all clarified wells was coated and cultured in BHI agar at 28°C and the lowest concentration of thymol with no bacterial growth after 24 h was considered as MBC (Vu et al., 2016). All experiments were repeated three times.

The effects of different concentrations of thymol on the growth curve of *S. iniae* were determined by ultraviolet spectrophotometry. Referring to the method with slight modifications (Liu et al., 2020), *S. iniae* was diluted to 10^7^ CFU/mL and cultured in fresh BHI broth at a ratio of 1% (v/v). Different concentrations of thymol (0, 8, 16, 32, 64, 128, 256, and 512 μg/mL) dissolved in DMSO were added to each culture flasks when the optical density of the culture reached 0.3 at 600 nm (*OD*_600_ nm = 0.3). The *S. iniae* was incubated at 28°C and 150 rpm, and samples were periodically taken from the solution to determine *OD*_600_ nm from 0 to 24 h. The same volume of DMSO was used as the control group and all experiments were repeated three times.

### Electron Microscope Observation

The effect of thymol on the morphology of *S. iniae* was determined by scanning electron microscopy (SEM) and transmission electron microscopy (TEM). The test bacterial cells grown to exponential-phase were treated with thymol at the concentration of 256μg/mL for 4, 8, and 16 h at 28°C. The samples were processed according to previously published methods (Zhang et al., 2015). Briefly, the bacterial pellet was obtained by centrifugation at 4,500 rpm for 10min and washed 3 times with sterile PBS, and then pre-fixed with 2.5% glutaraldehyde at 4°C. SEM samples were observed under SEM after dehydration in a series of graded alcohols (30, 50, 70, 80, 90, 95, and 100%) and vacuum plating. On the other hand, TEM samples were post-fixed with osmium tetroxide, dehydrated in acetone, embedded, sliced, and stained with uranyl acetate and lead citrate. Sections were examined with JEM-1400-FLASH Transmission Electron Microscope. The Cells treated with the same volume of DMSO for the same time were used as the control group.

### Conductivity Determination

The change of the conductivity of *S. iniae* was measured by a conductivity meter to reflect the change of the cell membrane permeability. Thymol at 256μg/mL was added to the exponential-phase *S. iniae* solution, followed by incubation in a shaker (28°C, 150 rpm). One milliliter of the mixture was removed at 0, 1, 2, 4, 6, and 8 h, and the supernatant was collected by centrifugation (4,500 rpm, 10min). A conductivity meter was used to measure the conductivity of the supernatant diluted 20 times with 5% sterile glucose solution (Yin et al., 2020). The same volume of DMSO was used as the control group and the experiments were repeated three times.

### Lactate Dehydrogenase Activity Measurement

Thymol at a concentration of 2MIC was added to the pre-cultured *S. iniae* solution and incubated in a shaker (28°C, 150 rpm). The suspension was collected at 0, 1, 2, 4, 6, and 8 h, and the supernatant was obtained by centrifugation at 4,500 rpm for 10min. LDH activity was measured using an LDH assay kit (Jiancheng Bioengineering, Nanjing, China) according to the manufacturer's instructions after sonication (VCX750, Sonics, Newtown, CT). The final results were expressed in units/liter (U/L) and all experiments were repeated three times.

### Soluble Protein Content Determination

The soluble protein content of *S. iniae* after treated with or without thymol was detected by BCA protein assay kit and sodium dodecyl sulfate polyacrylamide gel electrophoresis (SDS-PAGE) (Yuan et al., 2019). Thymol with 2MIC concentration was added to the bacterial solution pre-cultured to the exponential phase and further cultured for 4, 8, 16, and 24 h. The cultures were centrifuged (4,500 rpm, 10min) and the bacterial pellet was washed three times with sterile PBS. Subsequently, cells resuspended in PBS were sonicated, and 40 μL of sonication solution was mixed and boiled with 10 μL 5-fold loading buffer for 10min. After a short centrifugation, the samples were used for SDS-PAGE analysis. In addition, the protein concentration in the solution was determined according to the manufacturer's instructions. The *S. iniae* cells treated with the same volume of DMSO were the control group.

### Measurement of DNA Leakage

The leakage of DNA into the supernatant was determined according to the modified method (Wu et al., 2017; Zhou et al., 2020). *Streptococcus iniae* grown to exponential-phase was centrifuged at 4,500 rpm for 10min and washed three times with sterile PBS. Bacterial cells were collected and re-suspended in PBS to obtain a suspension of 10^7^ CFU/mL and treated with thymol at a final concentration of 2MIC for 0, 1, 2, 4, 6, and 8 h at 28°C. The suspensions at different time points were centrifuged at 4,500 rpm for 10 min, and DNA content was measured by an ultra-micro UV spectrophotometer (NanoDrop OneC, Thermo Scientific, Waltham, MA). The same volume of DMSO was used as the control group and the experiments were repeated three times.

### RNA Sequencing and Transcriptomic Analysis

The antibacterial mechanism of thymol against *S. iniae* was further investigated by measuring the expression levels of RNA in thymol-treated or untreated bacterial cells. *Streptococcus iniae* treated with 2MIC thymol for 8 h was selected for transcriptomic analysis based on our previous results and the cells treated with DMSO used as the control group. All data were analyzed on the online platform of Majorbio Cloud Platform (www.majorbio.com). The entire process included RNA extraction, RNA-seq library construction and sequencing, mapping reads to reference genome, expression analysis, identification of differentially expressed genes (DEGs), Gene Ontology (GO) annotation of DEGs and Kyoto Encyclopedia of Genes and Genomes (KEGG) enrichment analysis.

Total RNA of *S. iniae* was extracted according to the manufacturer's instructions (Invitrogen) of TRIzol reagent, and genomic DNA (gDNA) was removed by DNase I (TaKara). Then, the high quality of RNA (*OD*260/280 = 1.8~2.0, OD260/230 ≥ 2.0, RIN ≥ 6.5, and ≥100 ng/μL) was determined and screened by an Agilent 2100 Bioanalyzer. Ribosome RNA (rRNA) was removed from the samples by the Ribo-Zero Magnetic kit (epicenter), and then all mRNAs were fragmented by adding fragmentation buffer. Afterwards, double-stranded cDNA was synthesized using the SuperScript Double-Stranded cDNA Synthesis Kit (Invitrogen, CA) and the second strand cDNA with dUTP was recognized and degraded by UNG enzyme. Synthesized cDNA was end-repaired, phosphorylated, and “A” base added according to Illumina's library construction protocol. After quantification of the cDNA library by TBS380 (Picogreen), the paired-end RNA-seq sequencing library was sequenced with Illumina HiSeq × TEN.

After removing and discarding some low-quality reads, clean reads in each sample were obtained and mapped to the *S. iniae* reference genome (https://www.ncbi.nlm.nih.gov/genome/?term=Streptococcus+iniae) by Bowtie2. The expression levels of genes were calculated by the method of transcript per million (TPM), and the differentially expressed genes between the two groups were identified by statistical tools of DEseq2. The screening threshold of DEGs was *p*-adjust < 0.05 and |log_2_FoldChange| >1. When unigenes met the above conditions, the genes were considered to be significantly different, which could be used for GO annotation and KEGG pathway enrichment analysis in the library.

To characterize DEGs covering the three domains of Cellular Component (CP), Molecular Function (MF) and Biological Process (BP), Goatools was used to identify statistically significantly enriched GO terms using Fisher's exact test (Zhang et al., 2020b). The purpose of performing FDR correction is to reduce the false positive rate by bonferroni, Holm, BY, BH (multiple hypothesis test method). Afterwards, KOBAS 2.0 was used to identify statistically significantly enriched KEGG pathways using Fisher's exact test, which could identify the most important biological metabolic pathways and signal transduction pathways that DEGs involved in within *S. iniae*.

### Validation of DEGs by RT-qPCR

Eight random genes (4 up-regulated genes and 4 down-regulated genes) were selected for RT-qPCR verification according to RNA-seq results, and *16S rRNA* was used as the housekeeping reference gene to determine the relative expression of target genes. Primer 5.0 was used to design the required primers based on the obtained gene fragments (shown in Table 1), and the primers were synthesized by Sangon Biotech Co., Ltd. (Shanghai, China). The methods of extraction, purification, quality detection and reverse transcription of *S. iniae* RNA were described previously.

**Table 1:** Primer sequences for RT-qPCR.

**Gene**	**Description**	**Primer sequences (5′-3′)**	**Production length (bp)**
*dprA*	DNA-protecting protein DprA	F: ATGGCGTTCTGGTAGTCTAATC	101
		R: CTCCCTGCGACTGTTTGT	
*RS07840*	aldo/keto reductase	F: CCGTTGAAGCTGGCCTTATTA	99
		R: TAACTGCTGGCTTGATCTTGG	
*RS09090*	ABC transporter permease	F: GAACCATCATAGGAACCACCTC	100
		R: CCTGGTGTCCCTTTAACAACTA	
*RS07145*	branched-chain amino acid ABC transporter permease	F: CTCTGGAACATTGGGTCCTTAT	101
		R: GTCCTGAGAAGCCAAGAACA	
*rpoB*	DNA-directed RNA polymerase subunit beta	F: TACAGTTGCACAGGCTAATTCT	102
		R: CTAGCTGGGAACTCTTGGTTATT	
*ftsZ*	cell division protein FtsZ	F: GACAGGTGTCCGTCAAGATAAA	98
		R: CATATTGAGCACCTGCTTGTTG	
*pfkA*	6-phosphofructokinase	F: CTGGTTTAGTTGCCGGTGATA	99
		R: CAGGATAGCGAGCAGAATATAGG	
*secA*	preprotein translocase subunit SecA	F: ACCGTCGTGATGTGATTACTG	102
		R: TACTTCTAGCATGGGCCTCTA	
*16S rRNA*	16S ribosomal RNA	F: GAGAAGAACGGTAATGGGAGTG	108
		R: ACGCTCGAGACCTACGTATTA	

*F, Forward primer; R, Reverse primer*.

Ten microliter PCR system contained 5 μL of ChamQ Universal SYBR qPCR Master Mix, 3 μL of DNase/RNase-Free Water, 0.5 μL of forward and reverse primers, and 1 μL of cDNA. PCR conditions were initial step (95°C, 3min), 40 cycles (95°C, 10 s and × °C, 30 s) with a temperature gradient from 65 to 95°C for melting curve analysis (Bio-Rad), and the 2^−Δ*ΔCT*^ method was used to calculate the relative expression levels of genes according to previous reports (Zhang et al., 2020b).

### Protective Experiment

After a week of adaptive rearing, a total of 60 channel catfish were randomly divided into three 100-L tanks, with 20 fish per tank. As previously described, *S. iniae* was re-suspended in 0.9% sterile saline to obtain a suspension with a concentration of 1.5 × 10^8^ CFU/mL. Each fish in the infection and prevention group was intraperitoneally injected with 100 μL of bacterial suspension, while the fish were intraperitoneally injected with an equal volume of 0.9% sterile saline used as control. All fishes in the prevention group were administered with 20 mg/kg of thymol by a gavage needle (once a day for 5 days) before *S. iniae* challenge (Dong et al., 2020). The fishes in the control group and challenge group were administered with the same volume of sterile saline by a gavage needle. Feeding was maintained twice a day at 9 a.m. and 5 p.m. during the trial. The dead channel catfish were counted every 24 h for 2 weeks.

### Effects of Thymol on Serum Non-specific Immune Indicators

Thirty weight-matched healthy channel catfish were allocated into 3 groups, and the fishes in each group were treated above. Serum of 3 fish in each group was used to detect the non-specific immune indicators at 0 and 48 h after *S. iniae* infection, respectively. Blood was collected from the caudal vein of anesthetized fish (100 ppm of tricaine methanesulphonate, MS-222, Sigma-Aldrich, St. Louis, USA) using sterile syringe, and serum samples were obtained using centrifugation (6,500 rpm, 4°C, 10 min) and stored at −20°C until use (Ma et al., 2021). The activities of enzymes in serum including SOD, CAT, LZM, and ACP were determined using the relevant kits according to the manufacturer's instructions (Nanjing Jiancheng Bioengineering Institute, Nanjing, China). All experiments were repeated three times and the results were expressed as mean ± standard deviation (SD).

### Localization *S. iniae* in Tissues

Detection of *S. iniae* in different tissues of channel catfish in infection group and prevention group using real-time quantitative PCR. The required primers were synthesized according to the results of Corral and Santos (2021) (Forward primer, SilldP-F 5′-ACACAGGTGAGCACGCTAAA−3′, and reverse primer, SilldP-R 5′- CGTCACCATCGTCTTGGTCA−3′), and the amplified fragment was 167 bp. The genomic DNA of *S. iniae* was extracted according to the bacterial genomic DNA extraction kit (TIANGEN BIOTECH, Beijing, China), and the ChamQ Universal SYBR qPCR Master Mix was used for PCR amplification. The purified product was serially diluted 10-fold for real-time PCR amplification in order to establish a standard curve.

Each fish in the prevention group was given 20 mg/kg thymol solution (one per day for 5 days) according to the same method. Afterwards, the channel catfish in the infection group and the prevention group were transferred to a 100-L water tank, which contained *S. iniae* at a final concentration of 1.5 × 10^9^ CFU/mL, and all fish were re-transferred to the original circulating water system after 30min. Liver, spleen, blood, brain, gill, and intestine samples of 3 fishes from each group were collected with sterile conditions after 4, 8, 12, 24, 48, and 72 h post-infection. All samples were weighed and homogenized with 1 g/mL of 0.9% sterile saline, and the genomic DNA was extracted according to the bacterial genomic DNA extraction kit (TIANGEN BIOTECH, Beijing, China). All samples were used as templates for real-time quantitative PCR amplification, and the Cq values of the samples were converted into the copy number of amplified products according to the standard curve. All experiments were repeated three times and the results were expressed as mean ± SD.

### Data Analysis

Statistical significance was analyzed with GraphPad Prism 8.0 software (GraphPad Software, La Jolla, CA) using the unpaired two tailed Student's *t*-test or deviation analysis. All experiments were repeated three times and all data were presented as mean ± SD. A value of *P* < 0.05 was considered statistically significant difference.

## Results

### Drug Resistance of *S. iniae* Strain HT

The antibiotic susceptibility results of *S. iniae* strain HT were shown in Table 2. Briefly, it had a broad resistance spectrum of resistant to various antibiotics such as amoxicillin, kanamycin, roxithromycin, cefotaxime, penicillin, enrofloxacin, roxithromycin mycin, florfenicol, polymyxin B and meropenem.

**Table 2:** Antibiotic susceptibility of strain HT.

**Antibiotic**	**Sensitivity**
Cefradine	S
Amoxicillin	R
Ampicillin	S
Kanamycin	R
Spectinomycin	I
Enrofloxacin	R
Tetracycline	S
Vancomycin	S
Roxithromycin	R
Imipenem	S
Cotrimoxazole	I
Cefotaxime	R
Penicillin	R
Carbenicillin	R
Amikacin	R
Neomycin	I
Florfenicol	R
Doxycycline	S
Polymyxin B	R
Clindamycin	S
Meropenem	R
Azithromycin	S

*S, susceptible; I, intermediate; R, resistance*.

### Thymol Inhibited the Growth of Strain HT

**Figure 1:**
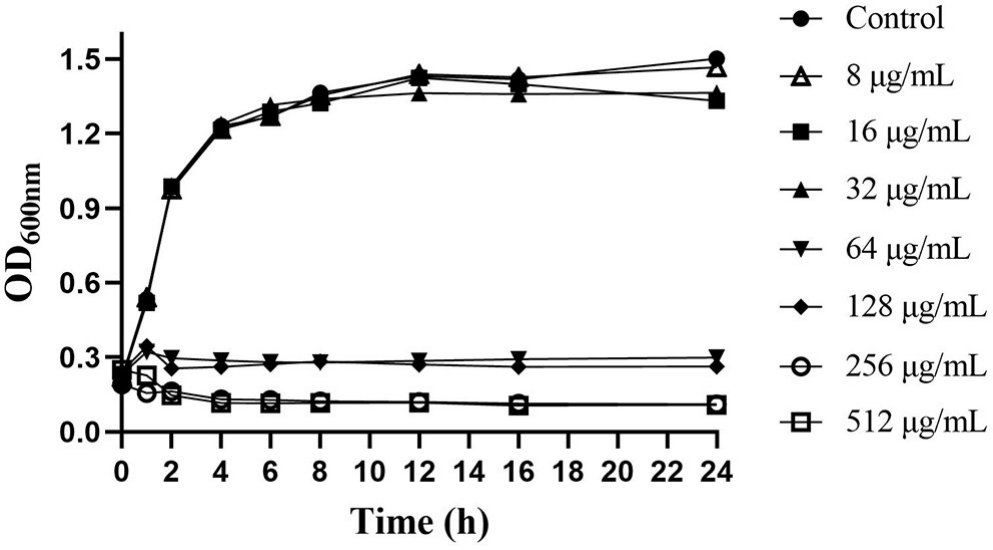
Effects of different concentrations of thymol on the growth of *S. iniae* strain HT in BHI broth.

Thymol had significant antibacterial activity against *S. iniae in vitro*, and the MIC and MBC values were 128 and 256μg/mL, respectively. The growth curves demonstrated that the concentration-dependent antibacterial effect of thymol against *S. iniae* (Figure 1). The control group was not observed growth inhibition, while the 8, 16, and 32μg/mL treatment groups had a slight inhibition. However, the growth of *S. iniae* was significantly inhibited with the concentration of MIC, 2MIC, and 4MIC thymol. Interestingly, at sub-inhibitory concentrations, thymol at 64μg/mL exhibited significant bactericidal activity after 2 h (Figure 1).

### Thymol Changed the Ultrastructure of Cells

**Figure 2:**
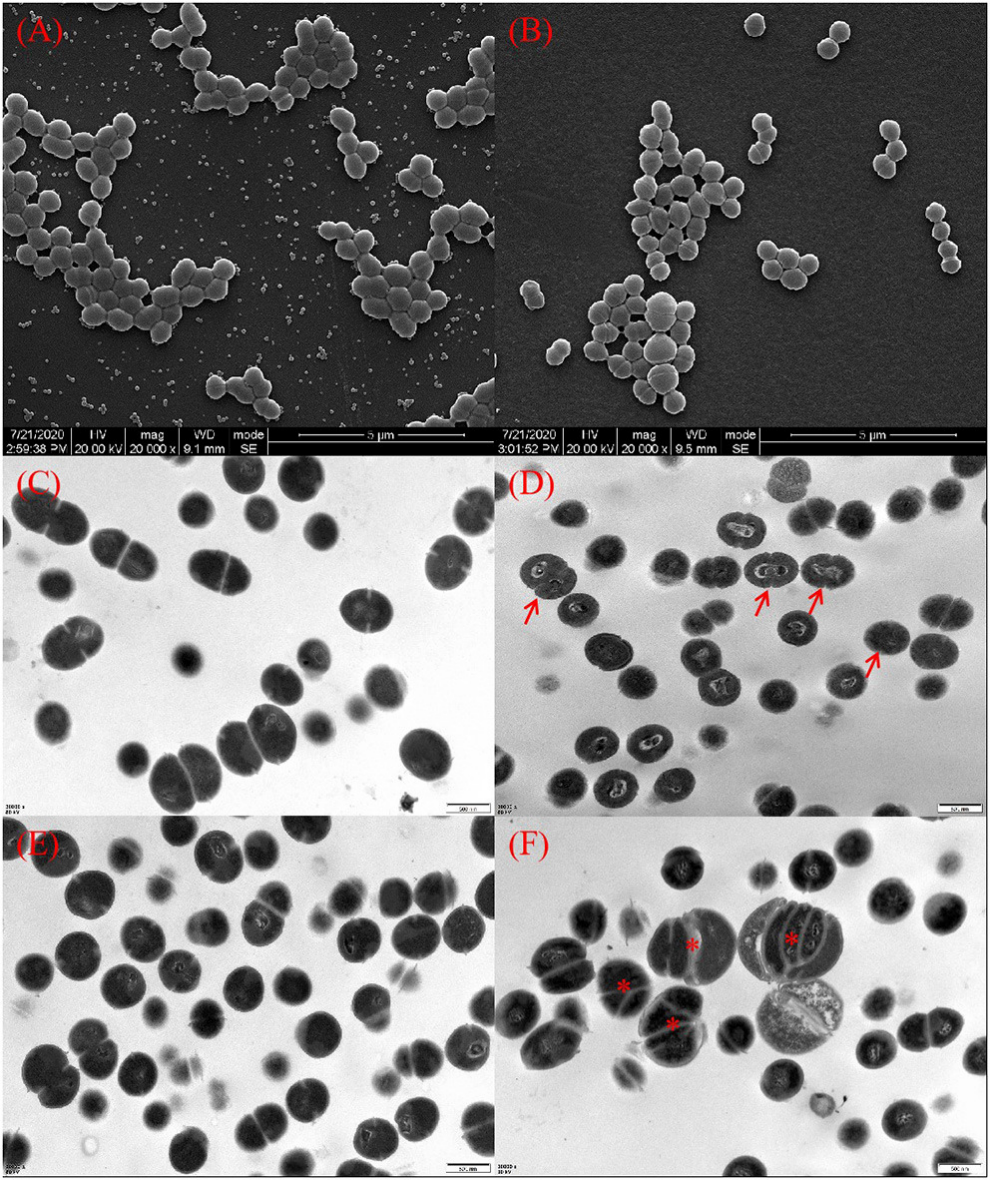
Morphology of *S. iniae* cells treated with or without thymol under SEM and TEM. **(A)** Untreated *S. iniae* after 4 h under SEM. **(B)** Thymol-treated *S. iniae* for 4 h under SEM. **(C)** Untreated *S. iniae* cells after 8 h under TEM. **(D)** Thymol-treated *S. iniae* for 8 h under TEM. The arrows indicate rough and wrinkled cell surfaces. **(E)** Untreated *S. iniae* cells after 16 h under TEM. **(F)** Thymol-treated *S. iniae* for 16 h under TEM. The asterisks indicated abnormal enlargement and division of cells.

SEM and TEM images of *S. iniae* treated with or without thymol were shown in Figure 2. Untreated bacterial cells had plump granules with smooth edges, spherical or oval, chain or row arrangement, and clear boundaries and could be observed under SEM (Figure 2A). However, cells in the treatment group were sparse, loosely arranged and irregular (Figure 2B). TEM images showed that the ultrastructure of cells in the thymol-treated group. The cells in the control group had complete cell membrane and cell wall, round and uniform staining, and normal binary fission (Figures 2C,E). Thymol treated cells showed damage, rough edges, blurred boundaries, and uneven staining (Figure 2D). After 16 h, the cell damage intensified, with abnormal enlargement and malformed fission, loss of cytoplasm, vacuolation and cell lysis (Figure 2F).

### Thymol Increased Cell Membrane Permeability

**Figure 3:**
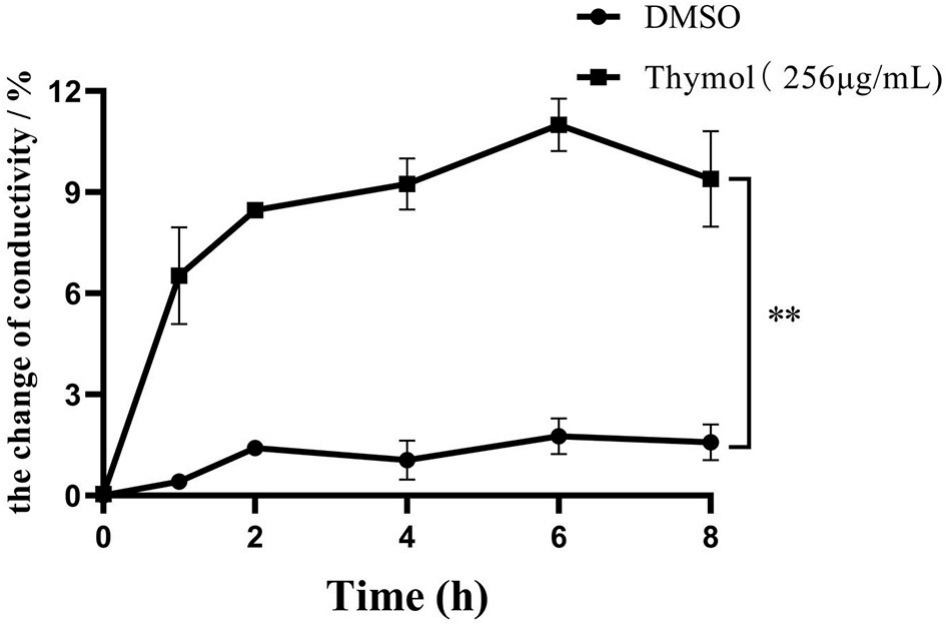
2MIC thymol increased the cell membrane permeability of *S. iniae*, resulting in increased conductivity in supernatant. (Thymol group vs. DMSO group, **p < 0.01).

The changes of conductivity reflect the increase of *S. iniae* membrane permeability and the results were shown in Figure 3. After 2MIC thymol treatment, the conductivity of HT strain continued to increase within 8 h, which was significantly different from the control group. The conductivity of the experimental group increased by 6.52±1.44% (*P* < 0.01) after 1 h, and reached the peak value of 10.99 ± 0.64% after 6 h (*P* < 0.01).

### Thymol Reduced the Concentration of Intracellular Protein

**Figure 4:**
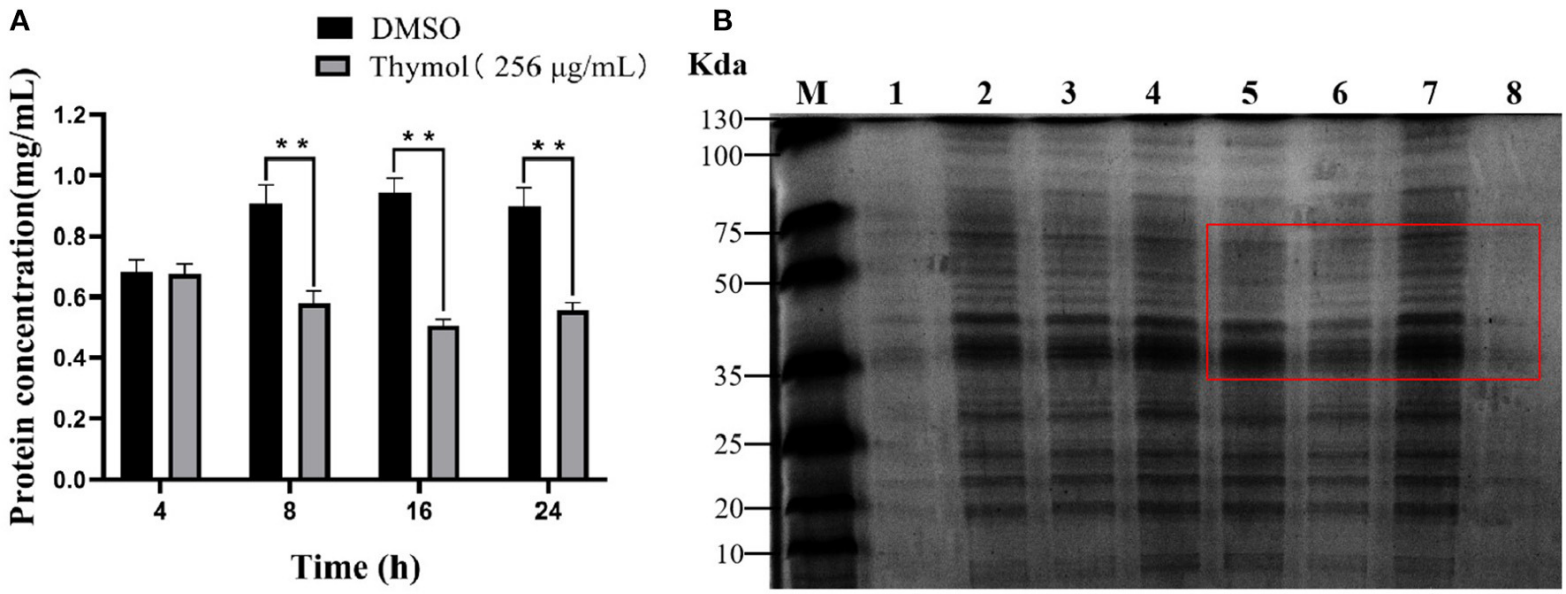
Effect of 2MIC thymol on soluble protein of *S. iniae* strain HT. **(A)** Thymol decreased the total protein concentration of HT **(B)** SDS-PAGE analysis results. Lane M, maker; lanes 1, 3, 5 and 7, untreated cells for 4, 8, 16 and 24 h, respectively; lanes 2, 4, 6 and 8, treated cells with thymol for 4, 8, 16 and 24 h, respectively. **p < 0.01 when compared with control group.

The protein results of *S. iniae* treated with or without thymol were shown in Figure 4. The protein concentration results indicated a time-dependent inhibitory effect of 2MIC thymol on the soluble protein of strain HT. The total protein concentration after 4 h of thymol treatment was similar to the control group. However, after 8, 16, and 24 h, the total protein concentration of the thymol group decreased to 0.58 ± 0.029, 0.50 ± 0.016, and 0.55 ± 0.02 mg/mL (*P* < 0.01; Figure 4A), respectively, which was significantly different from the untreated group. SDS-PAGE results showed clear and bright protein bands of HT strain in the control group (Figure 4B, lanes 1, 3, 5, and 7), however, the treatment group (Figure 4B, lanes 2, 4, 6, and 8) showed fewer and lighter bands than the control group. Changes in protein profile is time-dependent, which is consistent with the changes in protein concentration.

### Thymol Reduced LDH Activities of *S. iniae*

**Figure 5:**
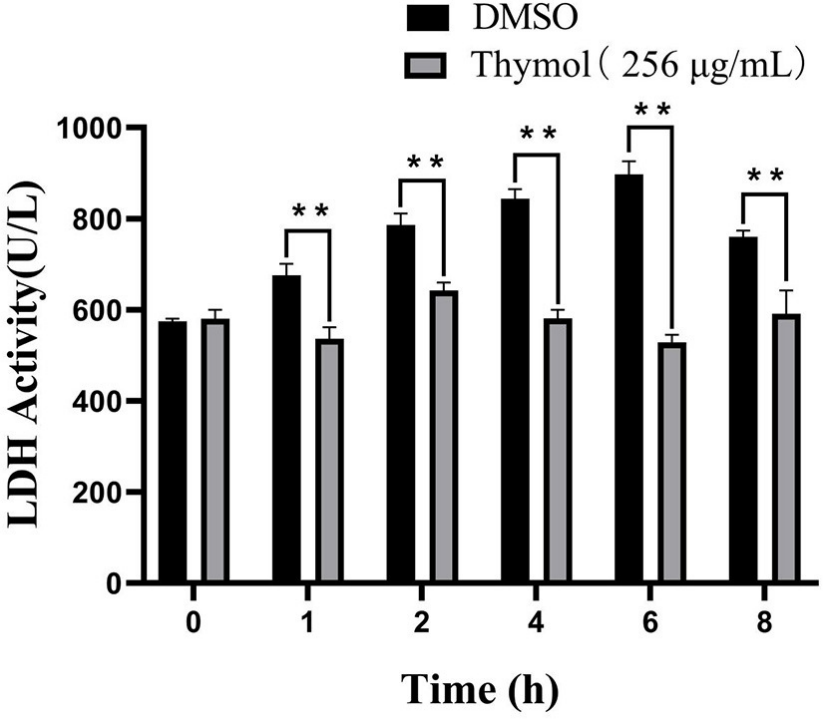
Thymol inhibited the LDH activities of strain HT with a time-dependent manner. **p < 0.01 when compared with control group at the same time point.

Thymol treatment had a negative effect on the LDH activity of *S. iniae* shown in Figure 5. LDH activities of the experimental groups decreased by 20.61, 18.25, 30.17, 41.11, and 22.22% after 1, 2, 4, 6, and 8 h compared with control group, respectively (*P* < 0.01). The results indicated that thymol could inhibit the LDH activity of *S. iniae*.

### Thymol Increased DNA Leakage

**Figure 6:**
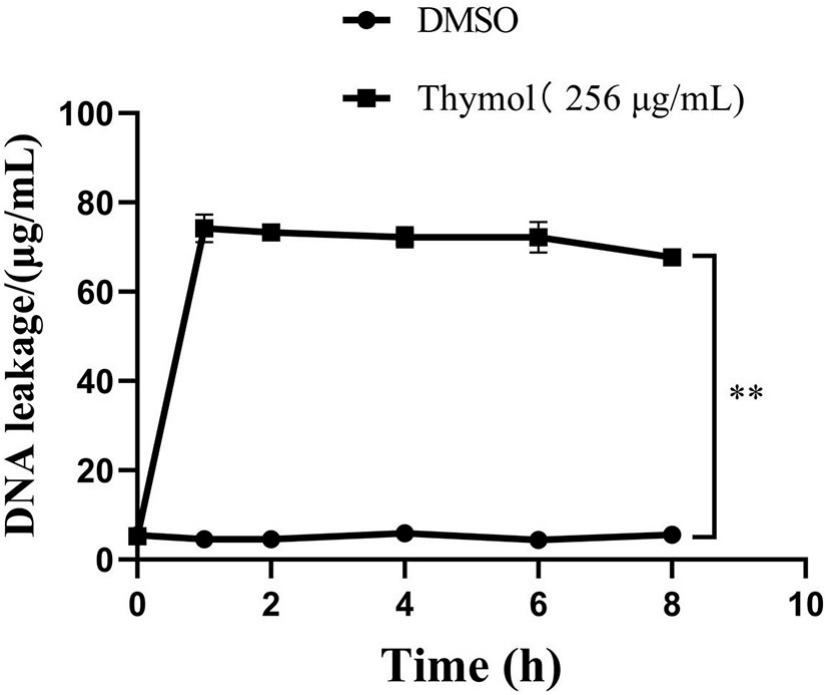
Thymol increased the leakage of *S. iniae* DNA. **p < 0.01 when compared with control group.

The results of DNA leakage were shown in Figure 6. In the control group, the concentration of DNA in the suspension did not change significantly and remained at 5μg/mL until 8 h. However, the DNA concentration in the supernatant of *S. iniae* treated with thymol increased rapidly to the peak value of 74.19 ± 2.54μg/mL (*P* < 0.01). Subsequently, although the value decreased gradually after 8 h, it was still higher than the control group by 62.1 ± 0.39μg/mL (*P* < 0.01).

### Transcriptome Sequencing and Assembly

To further explore the potential antibacterial mechanism of thymol against *S. iniae*, RNA-seq transcriptome analysis was performed on strain HT. Here, RNA-seq data from control *S. iniae* (no thymol treatment) and 2MIC thymol treatment for 8 h were compared. After low-quality reads were screened and filtered, 26,003,354, 29,702,112, and 28,336,922 clean reads were produced by Con 1, Con 2, and Con 3, while 22,671,456, 22,694,362, and 22,308,468 clean reads were produced by Exp 1, Exp 2, and Exp 3, respectively. The error rate of all clean reads was < 0.05% and the rate of Q30 was >94% (Table 3).

**Table 3:** Transcriptome data summary and quality.

**Sample**	**Raw reads**	**Clean reads**	**Clean bases (bp)**	**Error rate (%)**	**Clean Q20 (%)**	**Clean Q30 (%)**
Con 1	27,652,984	26,003,354	3,588,843,352	0.0244	98.39	94.66
Con 2	31,389,830	29,702,112	3,765,454,891	0.0236	98.68	95.48
Con 3	29,053,660	28,336,922	3,826,609,519	0.0241	98.49	94.86
Exp 1	23,734,268	22,671,456	2,827,387,863	0.0245	98.3	94.57
Exp 2	24,462,208	22,694,362	2,817,631,365	0.0243	98.38	94.74
Exp 3	23,850,156	22,308,468	2,863,700,316	0.0246	98.28	94.46

*Con 1, Con 2, and Con 3 were biological duplications in the control group, while Exp 1, Exp 2, and Exp 3 were duplications in the thymol treated group*.

### Sequence Mapping Result

The high-quality reads of the transcriptome were compared with the *S.iniae* reference genome sequence. The total genome mapping rates of the control group were 92.55, 83.83, and 91.98%, respectively, while the total genome mapping rates of the treatment group were 84.9, 83.02, and 78.12%, respectively. In addition, the percentages of clean reads to unique locations in the reference genome were 92.45, 83.68, and 91.88% in the control group, and 84.83, 82.92, and 78.01% in the treatment group, respectively (Table 4).

**Table 4:** Clean reads mapping with reference genome.

**Sample**	**Total reads**	**Genome mapped reads**	**Genome mapped ratio (%)**	**Unmapped reads**	**Unmapped reads ratio (%)**	**Uniquely mapped reads**	**Uniquely mapped reads ratio (%)**
Con 1	26,003,354	24,065,935	92.55	1,937,419	7.45	24,040,972	92.45
Con 2	29,702,112	24,899,009	83.83	4,803,103	16.17	24,854,372	83.68
Con 3	28,336,922	26,063,359	91.98	2,273,563	8.02	26,035,118	91.88
Exp 1	22,671,456	19,248,258	84.9	3,423,198	15.1	19,232,797	84.83
Exp 2	22,694,362	18,840,731	83.02	3,853,631	16.98	18,817,868	82.92
Exp 3	22,308,468	17,427,706	78.12	4,880,762	21.88	17,403,496	78.01

### Identification of DEGs

**Figure 7:**
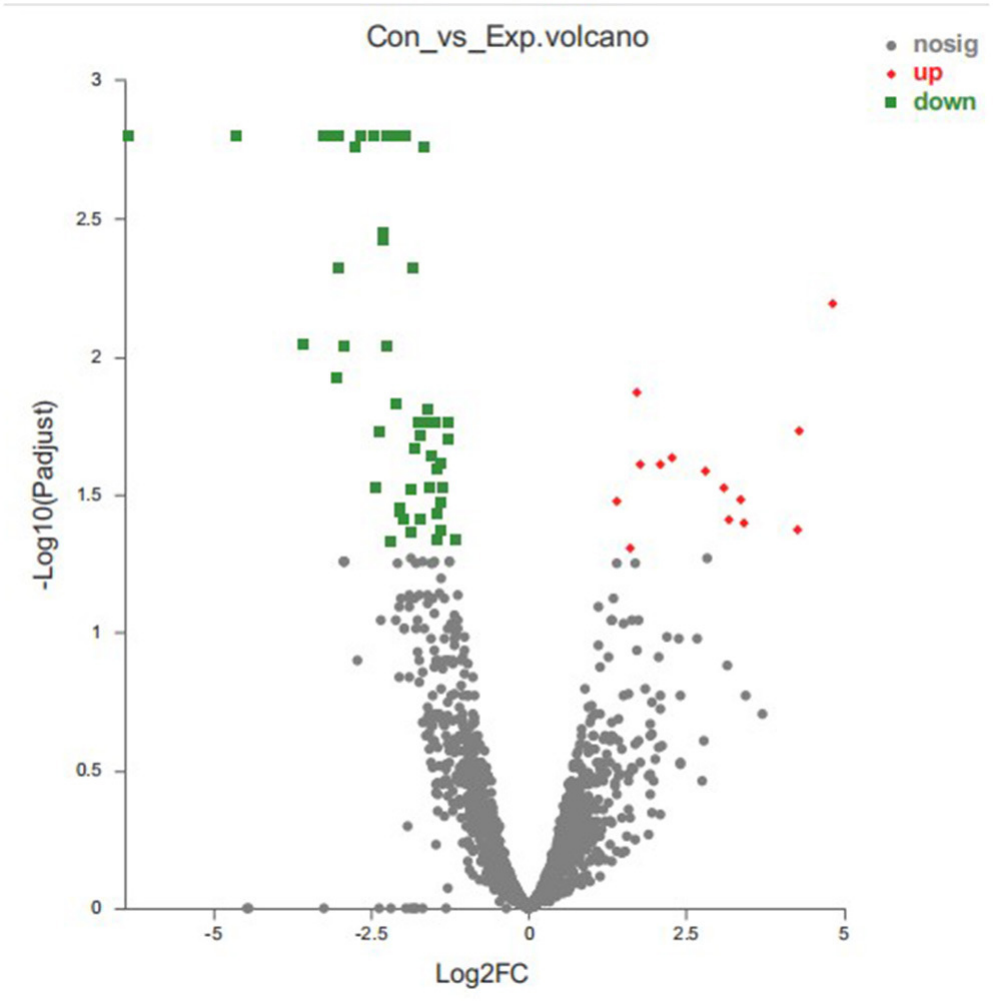
Statistics of differentially expressed genes. X-axis: fold change value (FC) of gene expression difference between two groups of samples; Y-axis: *p-adjust* value. Each dots represents a specific gene in this figure: Red points: up-regulated genes (13); Green points: down-regulated genes (51); Gray points: non-significantly different genes.

We identified 64 DEGs between control group and treatment group by using DEseq2 analysis software with *p*-adjust < 0.05 and | log_2_FoldChange | > 1 as the selection criteria, and the total DEGs contained 51 down-regulated genes and 13 up-regulated genes (Figure 7). Supplementary Table 1 showed the detailed description of DEGs between the thymol-treatment and the control groups.

### GO Annotation of DEGs

**Figure 8:**
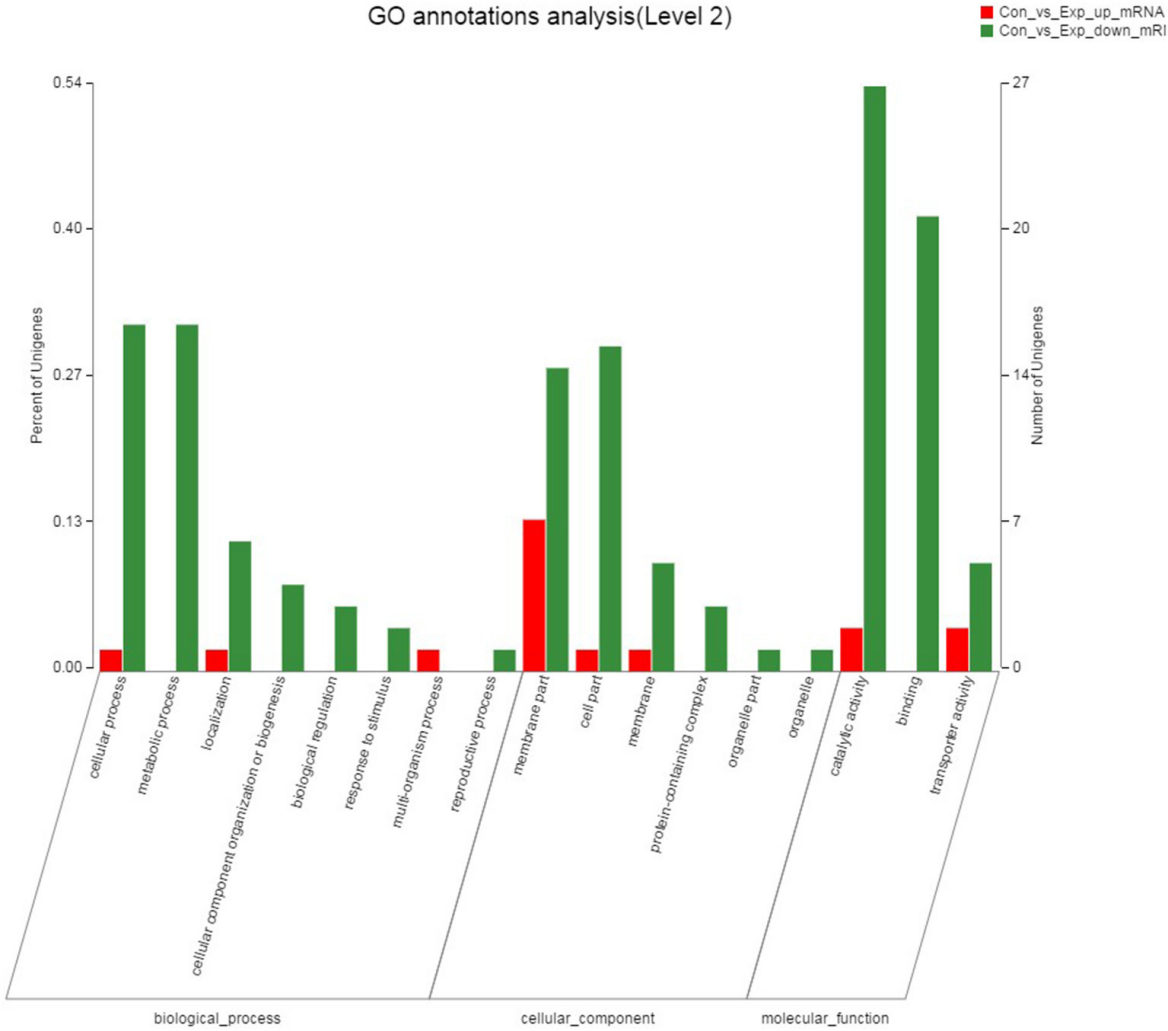
GO annotation analysis of DEGs between the control group and thymol treated group. The X-axis represents the GO terms, and the genes were annotated in three main categories: biological process (BP), cellular component (CC) and molecular function (MF). The Y-axis represents the number of genes for each term and the percentage of total differentially expressed genes, respectively. Each rectangle represents an annotated GO term. The colors represent up and down-regulation genes.

The result of the GO annotation for DEGs was shown in Figure 8. All DEGs were assigned into 17 GO terms. Catalytic activity (GO: 0003824), binding (GO: 0005488) and membrane part (GO: 0044425) were the three most important functional groups, with 29, 21, and 21 differentially expressed genes annotated in these GO terms, respectively. In biological processes category, cellular processes (GO: 0009987) and metabolic processes (GO: 0008152) were the most abundant GO terms, and membrane part (GO: 0044425) and cellular part (GO: 0044464) were the most common subcategories of cell components. GO annotation results indicated that the antibacterial mechanism of thymol may be associated with the destruction of the cell membrane of *S. iniae*.

### KEGG Enrichment Analysis of DEGs

**Figure 9:**
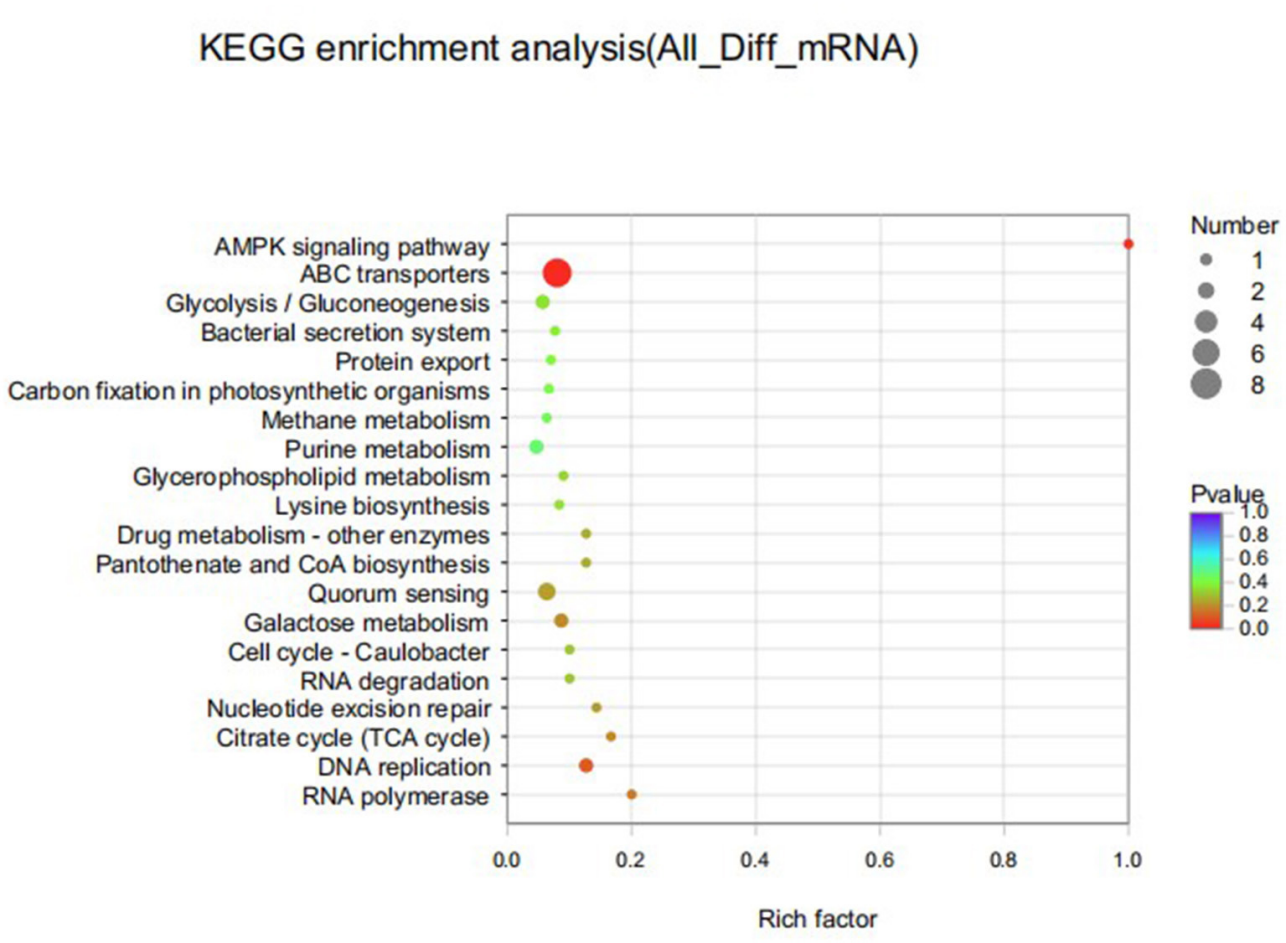
Top 20 KEGG pathways enriched for differentially expressed genes of *S. iniae* strain HT after treatment with thymol. The X-axis represents Rich factor. The Y-axis represents KEGG pathway entry.

KEGG significant enrichment analysis was performed on the differentially expressed genes to obtain the main pathways and biological functions involved in DEGs, and the KEGG pathway enrichment results were shown in Figure 9. In the top 20 enriched pathways, DEGs were significantly involved in ABC transporters, DNA replication and AMPK signaling pathways. There were 9 pathways related to metabolism, 6 pathways related to genetic information processing, 3 pathways related to environmental information processing, and 2 pathways related to cellular processes. Furthermore, down-regulated DEGs were significantly enriched in D-glutamine and D-glutamate metabolic pathways, while up-regulated DEGs were mainly involved in ABC transporters, quorum-sensing and pantothenate and CoA biosynthesis pathways. There were 8 DEGs in ABC transporter pathway with both up and down regulated.

### Validation of DEGs by RT-qPCR

**Figure 10:**
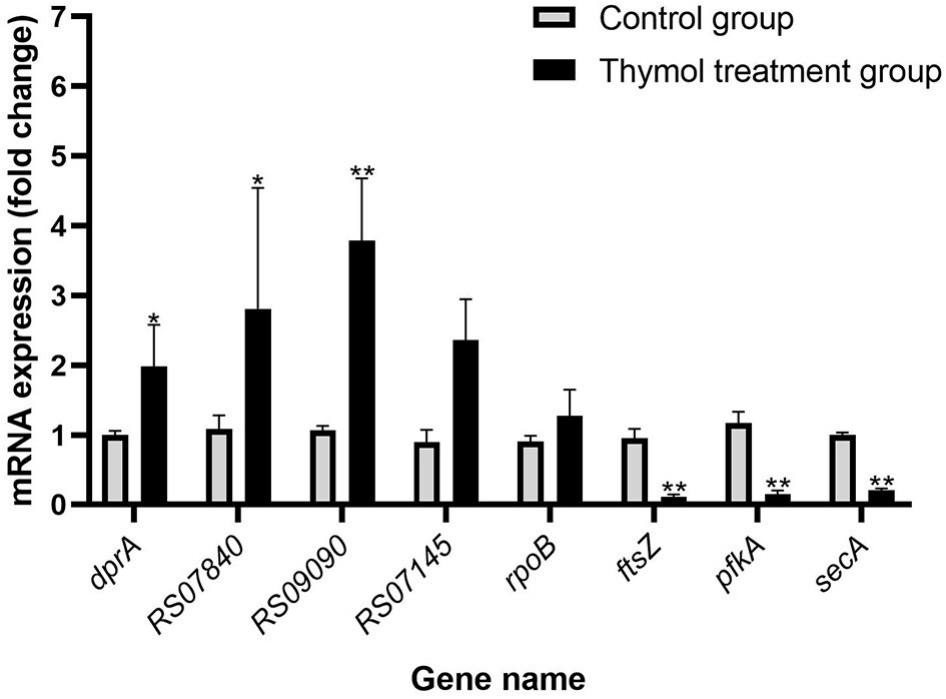
Validation of genes in the two groups by RT-qPCR. All experiments were repeated three times and the results were expressed as mean ± SD. The symbol ** represents an extremely significant difference (*P* < 0.01); the symbol * represents a significant difference (*P* < 0.05).

In order to verify the validity of transcriptome results, 4 genes with up and down-regulated DEGs were randomly selected for RT- qPCR. The relative expression levels of DEGs were similar to the RNA-seq data, demonstrating the validity of transcriptome analysis (Figure 10).

### Thymol Protected Channel Catfish From *S. iniae* Infection

**Figure 11:**
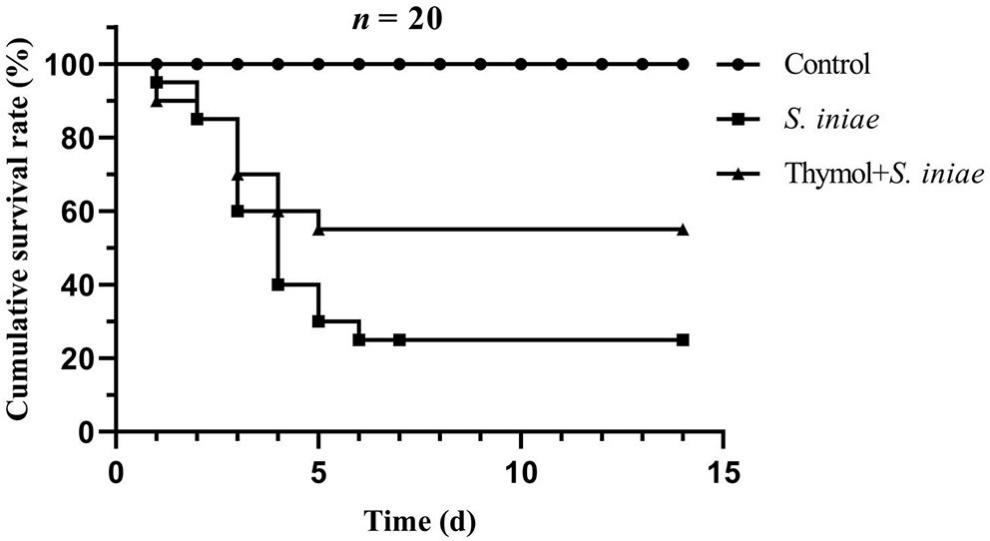
The cumulative survival rate of channel catfish in each group after 2 weeks post-infection.

The cumulative survival rate of each group was shown in Figure 11. In the control group, there were no death and symptoms on the body surface, and the fish activity and feeding were normal. However, the channel catfish in the infected group began dying 1 day after the *S. iniae* challenge, and showed a peak of mortality on days 3 and 4 post-infection. Most of the fish died acutely had no obvious symptoms on the body surface, but some fish had bleeding spots on the body surface and fins, and the infected fish fed less and swam on the surface. The cumulative mortality was 75% in the infection group until 15 days, while 45% in the prevention group. The results of the protection experiment indicated that thymol was beneficial for channel catfish against drug-resistant *S. iniae* infection.

### Thymol Increased the Activity of Non-specific Immune Enzymes

**Figure 12:**
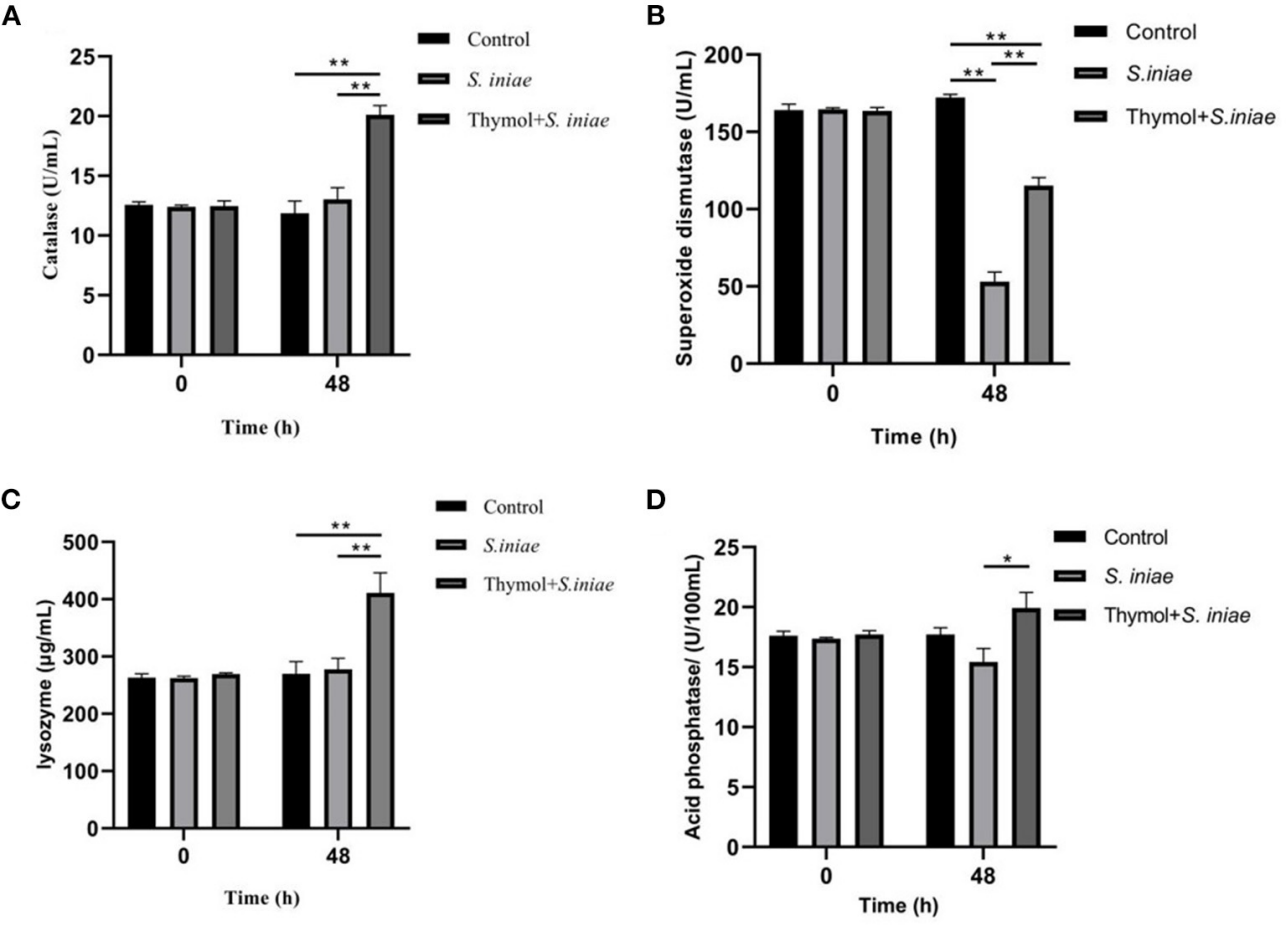
Effects of thymol on serum non-specific immune indicators in channel catfish. **(A)** CAT activities; **(B)** SOD activities; **(C)** LZM activities; **(D)** ACP activities. And all experiments were repeated three times and the results were expressed as mean ± SD. The symbol ** represents an extremely significant difference (*P* < 0.01); the symbol * represents a significant difference (*P* < 0.05).

SOD, CAT, and LZM were detected as antioxidant indicators, and ACP was detected as marker of lysosome, and the results were shown in Figure 12. There was no significant difference in enzyme activity between the infection group and the prevention group before the *S. iniae* infection. After 48 h post-challenge, CAT activities increased to 20.11 ± 0.64 U/mL in the prevention group, which was significantly higher than that in the infection group (13.04 ± 0.78 U/mL) and the control group (11.86 ± 0.35 U/mL) (*P* < 0.01). The values in the infection group were slightly higher than those in the control group, but not statistically significant (Figure 12A). Similarly, LZM activities in the prevention group significantly increased to 410.85 ± 28.76μg/mL compared with the control group (*P* < 0.01), and there was no significant difference between the prevention group and the control group (Figure 12C). However, the SOD activities in the infection group and the prevention group was significantly lower than the normal level, which were 53.01 ± 5.03 U/mL and 115.26 ± 4.18 U/mL, respectively (*P* < 0.01; Figure 12B). Moreover, ACP activities in the prevention group were also higher than the infection group after 48 h post-challenge (*P* < 0.05; Figure 12D). The results showed that thymol can increase the non-specific enzymatic activity of channel catfish.

### Thymol Reduced the Localization of *S. iniae* in Tissues

**Figure 13:**
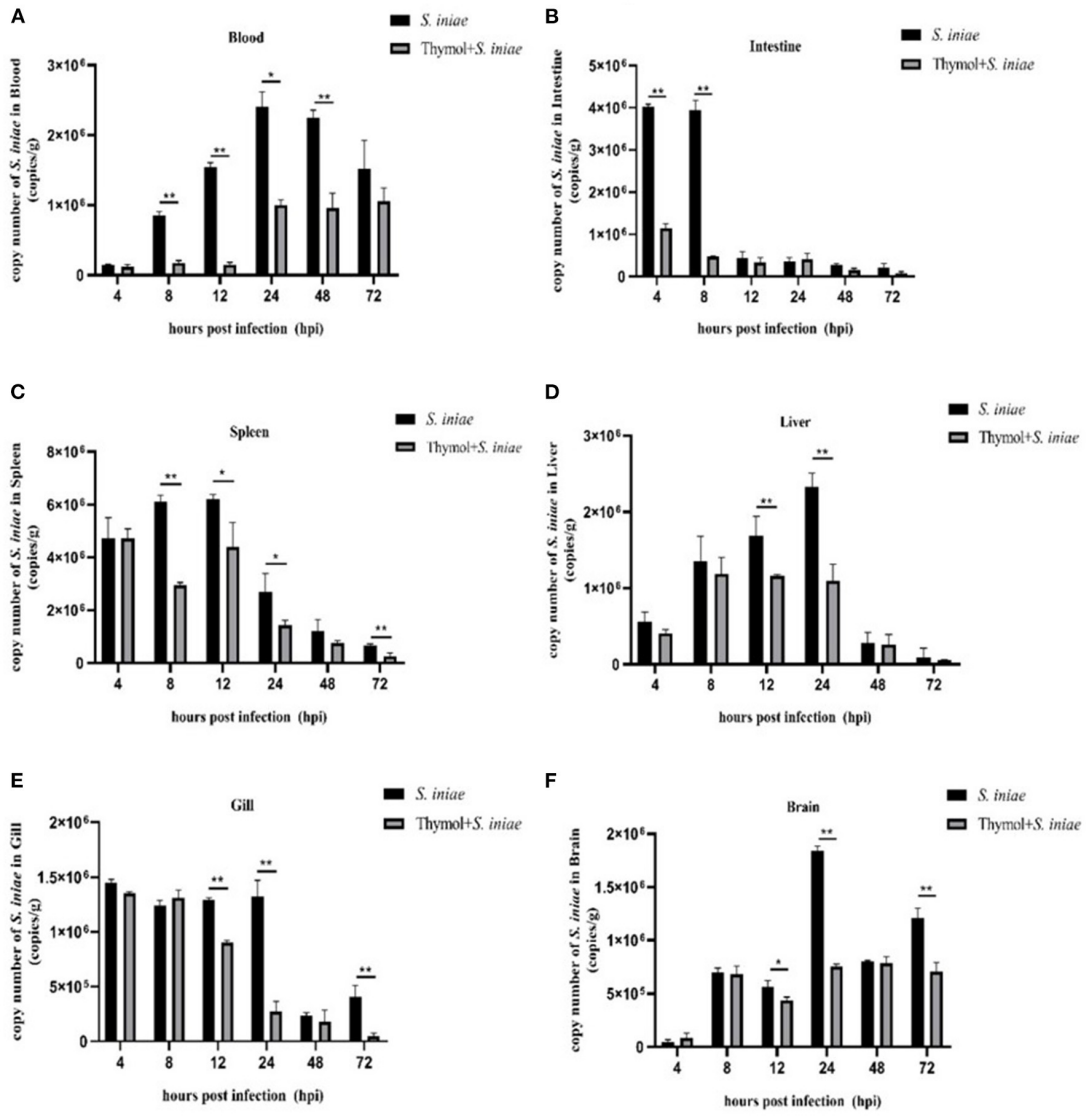
Copy numbers of *S. iniae* in tissues of channel catfish. **(A)** Localization of *S. iniae* in blood; **(B)** Localization of *S. iniae* in intestine; **(C)** Localization of *S. iniae* in spleen; **(D)** Localization of *S. iniae* in liver; **(E)** Localization of *S. iniae* in gill; **(F)** Localization of *S. iniae* in brain. The X-axis represents hours post-challenge and the Y-axis represents the number copies of *S. iniae* per gram of tissue. The symbol ** represents an extremely significant difference (*P* < 0.01); the symbol * represents a significant difference (*P* < 0.05).

Real-time quantitative PCR was used to detect the presence of *S. iniae* in tissues to verify the protective effect of thymol on channel catfish (Figure 13). *Streptococcus iniae* was detected in all samples, and thymol reduced the localization of *S. iniae*. The copy number of *S. iniae* in blood increased first and then decreased, and reached the peak after 24 h. Compared with the control group, the copy number in the thymol group decreased significantly (*P* < 0.01; Figure 13A). In the control group, the copy number of *S. iniae* in intestine was at a high level at 4–8 h, and then gradually decreased. The copy number of bacteria in the thymol group decreased by about 75% (*P* < 0.01; Figure 13B). The copy number of *S. iniae* in spleen of the thymol group was similar to that of the control group after 4 h, and was significantly decreased from 8 to 72 h (*P* < 0.05; Figure 13C). The change trend of the copy number in liver was similar to that in blood, which increased gradually before 24 h and decreased after 24 h. The results of the experiment group were significantly lower than those of the control group at 12–24 h (*P* < 0.01; Figure 13D). In gill, the number of *S. iniae* in the thymol group was significantly lower than that in the control group at 12, 24, and 72 h (*P* < 0.01; Figure 13E). There was a significant difference in the localization of *S. iniae* in brain between the thymol group and the control group at 24 and 72 h (*P* < 0.01; Figure 13F). The results suggested that thymol was beneficial for channel catfish against *S. iniae* infection.

## Discussion

Over the past decades, *S. iniae* was extremely pathogenic to dozens of aquatic animals and has become one of the most serious aquatic pathogens in the world. *Streptococcus iniae* can enter the bloodstream from a wound or digestive tract and eventually enter the central nervous system, so its infection was often accompanied by fatal meningoencephalitis (Agnew and Barnes, 2007). According to Aamri, *S. iniae* exists and uses phagocytes to evade the host's immune response (Aamri et al., 2015). It has also been suggested that serotype capsules confer *S. iniae* additional antiphagocytic properties (Locke et al., 2007). Soh et al. (2020) reported two virulence factors of *S. iniae*, extracellular nuclease (SpnAi) and secreted nucleotidase (S5nAi), which play important roles in the immune escape of *S. iniae*. Besides, biofilms seem to play an important role in resisting the host's immune defenses during *S. iniae* infection (Heckman and Soto, 2021).

Antibiotics and vaccines are the two main methods to preventing and controlling bacterial diseases. However, serological diversity and genetic variability limit the development and large-scale application of *S. iniae* vaccines (Agnew and Barnes, 2007). The abuse of antibiotics will lead to the enhancement of bacterial resistance and the emergence of multi-drug-resistant bacteria. In this study, *S. iniae* strain HT exhibited resistance to a variety of clinical antibiotics, which was consistent with the previous report by Feng et al. (2021). Besides, the irregular use of antibiotics and the lack of supervision lead to a series of problems, such as environmental pollution, drug residues in food and potential health hazards for human (Limbu et al., 2020). Therefore, there is an urgent need to find and develop green, safe and efficient methods to combat *S. iniae* infection.

Thymol has been used in traditional medicine because of numerous pharmacological properties including anti-inflammatory, antitumor, antioxidant, analgesic and antibacterial activities in many countries, and its safety was guaranteed as a natural plant compound (Rathod et al., 2021). There were a number of convincing studies have shown that thymol has a strong inhibitory effect on a variety of pathogens and highly resistant bacteria (Meeran et al., 2017; Salehi et al., 2018; Sepahvand et al., 2021). Therefore, we expect to develop thymol as a new environment-friendly drug to control *S. iniae* infection to replace traditional antibiotics.

In the present study, the potent antibacterial activity of thymol against *S. iniae in vitro* was evaluated based on MIC, MBC and growth curve. The underlying mechanism was explored by electron microscopy, nucleotides and proteins changes, and transcriptome analysis. Firstly, SEM and TEM results can directly reflect the changes of cell morphology and ultrastructure of *S. iniae*. Compared with the control group, the surface and edge of the cells in the treatment group were rough, obviously enlarged and the division was abnormal. According to Yuan's study, the arrangement of *S. aureus* treated with thymol was loose and irregular, and the intracellular material was lost, and cytoplasmic condensation under electron microscopy, indicating the destruction of cell wall and cell membrane (Yuan et al., 2019). On the one hand, Wang reported that thymol was concentration-dependent on cell wall and cell membrane damage in *S. aureus* (Wang et al., 2017). On the other hand, the matching of DNA leakage and conductivity results indicated an up-regulation of cell membrane permeability or cell membrane damage. Bacterial cell membranes have selectivity, participating in nutrient absorption, transport of intracellular proteins and small molecules, and the generation of drug resistance is essential for cell survival (Costerton and Cheng, 1975; Kamiura et al., 2019). Under normal physiological conditions, DNA is not allowed to pass through the cell walls and cell membranes, but the leakage of DNA indicates an increase of membrane permeability. The conductivity of *S. iniae* increased rapidly after thymol treatment, possibly due to the leakage of inorganic ions and macromolecules such as nucleotides and proteins in the cells. As a hydrophobic antibacterial agent targeting the plasma membrane, the effect of thymol on cell membrane permeability has been confirmed in a variety of bacteria (Wang et al., 2017; Qi et al., 2020; Yin et al., 2020; Liang et al., 2021). In addition, the soluble protein concentration of *S. iniae* treated with thymol was lower than that of the control group, and the inhibition was time-dependent, indicating that thymol inhibited the expression of soluble protein or promoted the loss of protein. Combined with the trend of DNA concentration in the supernatant, we believe that the decrease in soluble protein content is a strong evidence for the loss of membrane integrity. As an important glycolytic enzyme in bacterial cytoplasm, LDH plays an important role in maintaining virulence, forming bacterial biofilms and resisting host immunity (Market, 1984). The reduced in LDH activity confirmed the hypothesis of protein loss, indicating damage to cell walls and cell membranes. It has been reported that oregano essential oil disrupted the integrity of *Shewanella putrefaciens* cell membrane leading to the release of LDH into the medium, thus affecting the cytoplasm and glycolysis pathways of *S. putrefaciens* (Lan et al., 2022). Based on our previous results, thymol could significantly reduce the activity of LDH, the counts of biofilms and the virulence of *A. hydrophila* (Liang et al., 2021). Besides, thymol can insert and bind to DNA, affecting its normal function and causing bacteria death (Wang et al., 2017; Liu et al., 2021). In recent years, more and more reports have been reported on the antibacterial activity of natural compounds against *S. iniae*, and various antibacterial mechanisms have been explored. Chitosan, a natural alkaline polysaccharide, has excellent bactericidal activity by binding to the surface of *S. iniae* cells, altering their permeability and causing leakage of cytoplasmic components, ultimately leading to cell death (Beck et al., 2018). *Rosmarinus officinalis* and *Zataria multiflora* essential oils can reduce the hemolytic activity of *S. iniae* in a dose-dependent manner by inhibiting the expression of encoding gene sagA (Soltani et al., 2014). Flavonoids isolated from *Maclura tricuspidata* have potent antibacterial activity due to disruption of the cytoplasmic membrane and cellular structure of *S. iniae* (Lim et al., 2021).

Based on the present study, *S. iniae* cells treated with 2MIC thymol for 8 h were considered as the suitable samples for transcriptomic analysis. Total of 64 differentially expressed genes, including 51 down-regulated genes and 13 up-regulated genes were identified. The gene *ftsZ*, which encodes a cell division protein, was significantly down-regulated after thymol treatment for 8 h. The FtsZ protein was a highly conserved bacterial tubulin homolog that was mainly involved in cell division. FtsZ is the most important protein in the filamenting temperature-sensitive mutant (FTS) protein family, which first reaches the dividing position and recruits others relevant protein to form the cell wall between two new cells in most bacteria except *Streptococcus pneumoniae* (McQuillen and Xiao, 2020). In this study, the abnormal enlargement and division of *S. iniae* after thymol treatment may be related to the suppressed expression of *ftsZ* gene, indicating that the decrease of FtsZ protein content makes cells unable to complete division. Molecular docking and simulation studies showed that *Pennisetum typhoides* microgreens inhibited the FtsZ protein of *S. aureus*, according to the results of Sharma and Gupta (2021).

Furthermore, we found that streptolysin S (SLS) family TOMM toxins were significantly reduced after thymol treatment. SLS, one of the virulence factors of *Streptococcus*, was a small pore-forming cytotoxin with a wide range of membrane targets designated as a member of thiazole/oxazole-modified microcin (TOMM) group of natural products (Molloy et al., 2015). Locke found that SLS was an important virulence factor of *S. iniae*, and the loss of its production would significantly marked virulence attenuation (Locke et al., 2007). Our results suggest that thymol inhibits the expression of SLS-related genes, thereby reducing the virulence of *S. iniae*.

Glutamate racemate (*racE*) plays an important role in bacterial growth and was responsible for converting L-glutamate to D-glutamate, an important precursor for peptidoglycan synthesis (Mehboob et al., 2009). Muhammad et al. (2020) demonstrated that the presence of glutamate racemase effectively prevented D-glutamate starvation and cell wall destruction in *S. iniae*. In the present study, *racE* was down-regulated after thymol treatment, meaning that thymol can disrupt the integrity of *S. iniae* cell wall.

IMP dehydrogenase (*guaB*) plays an important role in the biosynthesis of guanine nucleotide, mainly catalyzing IMP to produce guanine nucleotide (GMP) precursor xanthosine monophosphate (Zhang et al., 2020a). The down-regulated *guaB* level suggested that inhibition of nucleotide biosynthesis may also be one of the mechanisms of thymol against *S. iniae*. In *Lactococcus lactis*, the expression level of *guaB* expression was closely related to growth rate and mortality (Ryssel et al., 2013).

Through the analysis of DEGs, we found that thymol inhibited glucose metabolism and transport pathways of *S. iniae*. 6-phosphofructokinase, fructose-6-phosphate aldolase and sugar-phosphate isomerase were important catalysts in glycolysis, while phosphoenolpyruvate carboxykinase was a key enzyme to complement oxaloacetate in TCA, which plays an important role in energy metabolism of the body. The phosphotransferase system (PTS) was a highly conserved transport mode of bacteria, in which more than 20 carbohydrates were transported and distributed, and plays a critical role in bacterial carbon storage and metabolism (Kotrba et al., 2001; Houot et al., 2010). The down-regulation genes indicated that limited energy metabolism of *S. iniae*.

Similarly, the transport systems of divalent metal cations including cadmium (*cadA*), and iron (*fetB*) of *S. iniae* appear to be inhibited by thymol due to the reduction of membrane transport capacity. Metal ions were necessary for the survival and growth of bacteria. They participate in the function of bacterial metabolism and various virulence factors, and contribute to the infection of pathogen microorganisms (Schalk and Cunrath, 2016). Bacteria have multiple mechanisms for transporting metal ions, and both the P-type ATPases and the ATP-binding cassette (ABC) family belong to ATP-driven efflux pumps (Agranoff and Krishna, 1998). P-type ATPases, distributed in the plasma membranes and named for phosphorylated aspartase enzyme intermediates, have been shown to be involved in the absorption and export of zinc ions in bacteria (Hantke, 2001). The down-regulated cadmium-translocating P-type ATPase and ATP-binding cassette protein may be a result of cell membrane damage and inhibition of energy metabolism in *S. iniae*.

In addition, DNA topoisomerase (*parE*), single-stranded DNA-binding protein (SSB), DNA primase (*dnaG*), excinuclease (*uvrB*), RNase, glycosyl hydrolase and DNA-directed RNA polymerase (*rpoB*) play important roles in DNA replication, recombination, repair and transcription pathways, respectively. We speculated that the down-regulation of related genes levels indicate that thymol may cause DNA damage and mutation in *S. iniae*, inhibit its repair and transcription, also affect the normal survival of cells.

The results of transcriptome sequencing showed that thymol affected a variety of biological functions of *S. iniae*, including inhibiting the transport of cell membrane, affecting the biosynthesis of cell wall, inhibiting the biosynthesis, interfering with the pathway of energy metabolism, reducing the expression of virulence factors and causing DNA damage. The results of RT-qPCR verification indicated the reliability of transcriptome data. However, *rpoB* displayed opposite results in RT-qPCR and DEGs analysis. According to Everaert et al., about 15% of genes showed inconsistent results between RNA-sequencing and qPCR data, and these genes were generally smaller, with fewer exons and lower expression (Everaert et al., 2017). In this study, the genes used for RT-qPCR verification were randomly selected, which may lead to inconsistent results between RNA-seq and RT-qPCR for *ropB*.

Cumulative survival rate, serum non-specific immune indicators and bacterial copies were used to determine the protective effect of thymol on channel catfish accompanied by *S. iniae* infection. The cumulative survival rate of the thymol prevention group was 55% and that of the infection group was 25%, suggesting that thymol can strengthen the constitution of the channel catfish and improve its resistance to pathogens. Thymol was widely used as an herbal feed additive for animals, poultry and fish, which can improve the growth and reproduction performance of livestock and poultry, scavenge free radicals, enhance immunity and improve intestinal microflora (El-hack et al., 2016; Alagawany et al., 2020). Meanwhile, it has been reported that thymol can improve the liver energy metabolism and antioxidant status of grass carp (Morselli et al., 2019).

On the other hand, thymol significantly increased the activities of superoxide dismutase, lysozyme, catalase and acid phosphatase in serum, indicating that thymol could enhance the non-specific immunity level of channel catfish. ACP was an important component of lysosomal enzyme in phagocytes and plays an important regulatory enzyme in animal metabolism. Its hydrolysis can destroy and degrade pathogens and plays a role in non-specific cellular immunity (Zhu et al., 2021). The ACP activities in the prevention group increased by 29.1% compared with the infection group (*P* < 0.05), which was beneficial for channel catfish to resist *S. iniae*. SOD was the key antioxidant enzyme of fish against free radical damage, which plays a crucial role in the balance between oxidation and antioxidant, and indicates the overall immune function of fish (Lin et al., 2014). Surprisingly, the SOD activities in the infection and prevention groups were lower than the control group. However, the values in the prevention group were significantly higher than that in the infection group, suggesting that the antioxidant level of channel catfish in the prevention group was relatively higher than that in the infection group (*P* < 0.01). LZM was widely found in fish mucus, organ secretions, serum and phagocytic lysosomes, and was indispensable in the process of anti-infection and immunity (Doan et al., 2019). The activities of LZM increased significantly in the prevention group, and the body needed it for increased resistance by hydrolyzing peptidoglycan and activating complement. Furthermore, CAT activities of the prevention group were significantly higher than that of the other groups, which was similar to the trend of LZM. CAT was an essential antioxidant enzyme in fish and the first line of defense against reactive oxygen species, thereby protecting organisms from oxidative stress (Gopi et al., 2019). Thymol has been shown to improve the physical condition and non-specific immunity levels of channel catfish, as previously described. Moreover, *Oliveria decumbens* can improve the resistance of Nile tilapia to *S. iniae* and up-regulate the activities of SOD and CAT. The dietary cecropin can improve the non-specific immunity of tilapia, including LZM, alkaline phosphatase (AKP), CAT and SOD (Lin et al., 2014).

Thymol reduced the copy number of *S. iniae* in tissues, indicating that the protective effect of thymol on channel catfish may be to reduce the invasion and colonization of pathogens. In addition, the copy number of bacteria in gills and intestines reached a peak after 4 h post-infection, which was speculated to be related to the mode of immersion infection.

## Conclusions

In this study, we found that thymol has potent antibacterial activity against drug-resistant *S. iniae*. Its potential antibacterial mechanisms were disruption of cell membrane and cell wall integrity, affect normal binary division, nutrient limitation, inhibition of metal ion transport, nucleotide biosynthesis and DNA repair and transcriptional pathways. In addition, *in vivo* studies have shown that thymol can reduce the cumulative mortality and bacterial load in tissues of *S. iniae*-infected channel catfish, and can significantly increase the activity of non-specific immune enzymes in serum. In conclusion, thymol is a reliable drug candidate against drug-resistant *S. iniae* infection.

## Data Availability Statement

The original contributions presented in the study are included in the article/Supplementary Material, further inquiries can be directed to the corresponding author.

## Ethics Statement

The animal study was reviewed and approved by Animal Care and Use Committee of Sichuan Agricultural University.

## Author Contributions

LY and PO contributed to the conception and method design of the study. CL, WW, SH, and YR participated in material preparation and experimental investigation. YG and WL performed data processing. CL contributed to result visualization. XH, CL, and DC wrote the first draft of the manuscript. SY, LY, and PO wrote sections of the manuscript. HG, JF, and HD participated in the review and revision of the manuscript. All authors have read and approved the submitted version of the manuscript.

## Funding

This work was supported by an Application Foundation Project of Sichuan Science and Technology Department (Grant Number 21YYJC2011) and Chengdu Science and Technology Project (2021-YF05-01672-SN).

## Conflict of Interest

The authors declare that the research was conducted in the absence of any commercial or financial relationships that could be construed as a potential conflict of interest.

## Publisher's Note

All claims expressed in this article are solely those of the authors and do not necessarily represent those of their affiliated organizations, or those of the publisher, the editors and the reviewers. Any product that may be evaluated in this article, or claim that may be made by its manufacturer, is not guaranteed or endorsed by the publisher.
