# Supplementary material for: The Antibacterial Activity of Thymol Against Drug-Resistant Streptococcus iniae and Its Protective Effect on Channel Catfish (Ictalurus punctatus)
Source: Front Microbiol. 2022 Jun 6;13:914868. doi: 10.3389/fmicb.2022.914868 (PMC9207766; doi:10.3389/fmicb.2022.914868)
Supplement: Supplementary file 1 [file Table_1.DOCX]

Supplementary Material

Supplementary Table 1 Differentially expressed gene list

| **Gene_id** | **Gene name** | **Gene description** | **Log2FC (Exp/Con)** | **Padjust** | **Regulate** |
| --- | --- | --- | --- | --- | --- |
|  |  |  |  |  |  |
| DIX59_RS00730 | *rpoB* | DNA-directed RNA polymerase subunit beta | -1.296279439 | 0.019829721 | down |
| DIX59_RS00835 | *-* | single-stranded DNA-binding protein | -2.755589021 | 0.001739836 | down |
| DIX59_RS00920 | *-* | glycyl-radical enzyme activating protein | -1.295307294 | 0.017377975 | down |
| DIX59_RS00935 | *-* | fructose-6-phosphate aldolase | -1.553806576 | 0.022905235 | down |
| DIX59_RS01070 | *-* | RpiB/LacA/LacB family sugar-phosphate isomerase | -3.063484115 | 0.011871463 | down |
| DIX59_RS01100 | *-* | PTS mannose/fructose/sorbose transporter family subunit IID | -1.712565831 | 0.019272339 | down |
| DIX59_RS01345 | *-* | hypothetical protein | 4.281902241 | 0.042385183 | up |
| DIX59_RS01635 | *ugpC* | sn-glycerol-3-phosphate ABC transporter ATP-binding protein UgpC | -1.144324872 | 0.046405504 | down |
| DIX59_RS01790 | *-* | GH92 family glycosyl hydrolase | -2.119550155 | 0.014795724 | down |
| DIX59_RS01820 | *-* | sugar ABC transporter permease | -2.174537678 | 0.046706351 | down |
| DIX59_RS01825 | *-* | carbohydrate ABC transporter permease | -1.964614001 | 0.038767386 | down |
| DIX59_RS01875 | *-* | NYN domain-containing protein | -1.854316621 | 0.004775157 | down |
| DIX59_RS02030 | *-* | phosphoenolpyruvate carboxykinase (ATP) | -1.456380936 | 0.025507703 | down |
| DIX59_RS02220 | *mecA* | adaptor protein MecA | -1.578414563 | 0.029875872 | down |
| DIX59_RS02585 | *-* | trypsin-like serine protease | -3.026763291 | 0.001586003 | down |
| DIX59_RS02670 | *-* | GNAT family N-acetyltransferase | -2.447052711 | 0.000239333 | down |
| DIX59_RS02830 | *ftsZ* | cell division protein FtsZ | -1.458781895 | 0.036893207 | down |
| DIX59_RS02955 | *cadA* | cadmium-translocating P-type ATPase | -3.002689108 | 1.70E-12 | down |
| DIX59_RS02960 | *-* | hypothetical protein | -2.65555278 | 0.000648767 | down |
| DIX59_RS03240 | *-* | bifunctional biotin--[acetyl-CoA-carboxylase] synthetase/biotin operon repressor | -2.234635813 | 5.47E-06 | down |
| DIX59_RS03450 | *-0* | ATP-binding cassette domain-containing protein | -1.366281739 | 0.029875872 | down |
| DIX59_RS03455 | *fetB* | iron export ABC transporter permease subunit FetB | -1.38732465 | 0.024176828 | down |
| DIX59_RS03540 | *uvrB* | excinuclease ABC subunit B | -1.87248147 | 0.043875408 | down |
| DIX59_RS03655 | *-* | DUF3042 family protein | 2.092864681 | 0.024720063 | up |
| DIX59_RS04195 | *-* | sugar transferase | -2.124732038 | 5.43E-05 | down |
| DIX59_RS04320 | *parE* | DNA topoisomerase IV subunit B | -1.565879011 | 0.017377975 | down |
| DIX59_RS04370 | *ssrA* | hypothetical protein | -4.635602816 | 5.46E-18 | down |
| DIX59_RS04400 | *pfkA* | 6-phosphofructokinase | -1.811109057 | 0.021270253 | down |
| DIX59_RS04660 | *-* | ABC transporter permease | -2.309417279 | 0.003566941 | down |
| DIX59_RS04690 | *coaC* | phosphopantothenoylcysteine decarboxylase | 1.405894992 | 0.033322629 | up |
| DIX59_RS04795 | *dprA* | DNA-protecting protein DprA | 2.815287484 | 0.02614163 | up |
| DIX59_RS04855 | *-* | sensor histidine kinase | -1.379294367 | 0.033760198 | down |
| DIX59_RS04865 | *xerS* | tyrosine recombinase XerS | -3.022868357 | 0.004775157 | down |
| DIX59_RS05780 | *dnaG* | DNA primase | -2.312069434 | 0.003757817 | down |
| DIX59_RS05805 | *-* | neutral zinc metallopeptidase | -2.178615825 | 0.000236567 | down |
| DIX59_RS05815 | *-* | ABC transporter permease | -1.494404964 | 0.017377975 | down |
| DIX59_RS05990 | *-* | streptolysin S family TOMM toxin | -6.36719515 | 9.27E-26 | down |
| DIX59_RS06245 | *-* | anti sigma factor C-terminal domain-containing protein | 1.76630079 | 0.02446279 | up |
| DIX59_RS06295 | *-* | YccF domain-containing protein | 4.830340525 | 0.006398348 | up |
| DIX59_RS06315 | *-* | ammonium transporter | 4.295344287 | 0.018674585 | up |
| DIX59_RS06625 | *-* | amino acid ABC transporter permease | -1.616044539 | 0.015493208 | down |
| DIX59_RS06660 | *dhaQ* | DhaKLM operon coactivator DhaQ | -1.960195887 | 0.000194727 | down |
| DIX59_RS07005 | *-* | DUF1275 domain-containing protein | 3.188239263 | 0.038767386 | up |
| DIX59_RS07145 | *-* | branched-chain amino acid ABC transporter permease | 3.355195625 | 0.032828071 | up |
| DIX59_RS07155 | *-* | ABC transporter substrate-binding protein | 3.087364792 | 0.029875872 | up |
| DIX59_RS07305 | *-* | segregation/condensation protein A | -3.252542833 | 0.000416833 | down |
| DIX59_RS07330 | *racE* | glutamate racemase | -2.046527258 | 0.035390542 | down |
| DIX59_RS07340 | *-* | diaminopimelate decarboxylase | -1.761702784 | 0.017377975 | down |
| DIX59_RS07415 | *-* | DnaD domain protein | -1.448859974 | 0.046405504 | down |
| DIX59_RS07490 | *-* | O-sialoglycoprotein endopeptidase | 1.620348594 | 0.04980524 | up |
| DIX59_RS07550 | *yqeH* | ribosome biogenesis GTPase YqeH | -3.027281451 | 1.28E-10 | down |
| DIX59_RS07670 | *rnpB* | RNase P RNA | -2.24583159 | 8.25E-06 | down |
| DIX59_RS07840 | *-* | aldo/keto reductase | 1.717807692 | 0.0134679 | up |
| DIX59_RS07910 | *rimP* | ribosome maturation factor RimP | -2.43249091 | 0.029875872 | down |
| DIX59_RS08240 | *-* | aldo/keto reductase | -2.380127294 | 0.018674585 | down |
| DIX59_RS08310 | *secA* | preprotein translocase subunit SecA | -2.273316573 | 0.009123096 | down |
| DIX59_RS08720 | *-* | MerR family transcriptional regulator | -1.868850056 | 0.030190221 | down |
| DIX59_RS09090 | *-* | ABC transporter permease | 3.410924773 | 0.0401774 | up |
| DIX59_RS09295 | *-* | isopeptide-forming domain-containing fimbrial protein | -1.567033639 | 0.029875872 | down |
| DIX59_RS09700 | *-* | FeoB-associated Cys-rich membrane protein | -3.578211607 | 0.008964184 | down |
| DIX59_RS09955 | *pgsA* | CDP-diacylglycerol--glycerol-3-phosphate 3-phosphatidyltransferase | -1.533078141 | 0.017377975 | down |
| DIX59_RS09995 | *guaB* | IMP dehydrogenase | -2.924712452 | 0.009142444 | down |
| DIX59_RS10175 | - | divalent metal cation transporter | -1.39013109 | 0.042385183 | down |
| DIX59_RS10245 | - | hypothetical protein | -1.650407938 | 0.001739836 | down |
